# Draft genome of a *Pseudovibrio* sp. isolated from the skeleton of *Pachyseris speciosa* from a Singaporean reef

**DOI:** 10.1128/mra.00763-24

**Published:** 2024-09-30

**Authors:** Aaron An Rong Loh, Dalong Hu, Jabez Mason Yong Jun Law, Elton Lim Wen Xiong, Lindsey Kane Deignan, Stephen Summers, Joao Paulo Andre Pereyra, Rebecca Josephine Case

**Affiliations:** 1 Singapore Centre for Environmental Life Sciences Engineering (SCELSE), Nanyang Technological University (NTU), Singapore, Singapore; 2 School of Biological Sciences, Nanyang Technological University (NTU), Singapore, Singapore; 3 Singapore Centre for Environmental Life Sciences Engineering (SCELSE), National University of Singapore (NUS), Singapore, Singapore; 4 Saw Swee Hock School of Public Health, National University of Singapore (NUS), Singapore, Singapore; 5 St John’s Island National Marine Laboratory c/o Tropical Marine Science Institute, National University of Singapore (NUS), Singapore, Singapore; University of Southern California, Los Angeles, California, USA

**Keywords:** corals, marine microbiology, *Pseudovibrio*

## Abstract

A *Pseudovibrio* sp. was isolated from the skeleton of the heat resilient coral *Pachyseris speciosa*. Genome analysis revealed the presence of the complete denitrification pathway and potential dimethylsulfoniopropionate metabolism which enhance coral resilience and production of tropodithietic acid, an antibiotic implicated in host defense.

## ANNOUNCEMENT

*Pseudovibrio* spp. belong to the family *Stappiaceae* (1) (class *Alphaproteobacteria*) (2) and are thought to be beneficial symbionts to marine invertebrates, such as sponges and corals (3). They can survive at a wide range of temperatures and fluctuating nutrients (4). They have the potential to act as probiotics in different marine systems due to the metabolites they produce, like tropodithietic acid (TDA), which inhibits *Vibrio* pathogens (5).

*Pachyseris speciosa* samples were collected from the seawall at the northern side of Kusu Island, Singapore, and maintained in an outdoor aquaria tank with a constant seawater flow. Samples were fragmented into ~9 cm^2^ (~2 g) fragments and ground with a mortar and pestle while mixing ~5 mL of sterile artificial seawater (macerate). Samples were treated with proteinase K, vortexed with glass beads, diluted with sterile artificial seawater, then spread plated on marine agar (Difco 2216) and incubated at 33°C until colonies were observed. Individual bacterial colonies were subcultured to ensure purity, and a single colony was used to inoculate marine broth for subsequent processing and sequencing.

Genomic DNA extraction used the DNeasy Powersoil Pro Kit (Qiagen, Hilden, Germany). DNA library preparation used the Accel-NGS 2S Plus Low Input DNA Library Prep Kit (Swift Biosciences, Ann Arbor, USA) before sequencing using an Illumina HiSeq X Ten platform, version 2.5 (Illumina, San Diego, CA, USA), generating 150-bp paired-end reads. DNA library preparation and sequencing were performed at Singapore Centre for Environmental Life Sciences Engineering, Nanyang Technological University.

Paired-end reads were trimmed using Trimmomatic, version 0.39 (6). Default parameters were used for all software unless specified. Genome assembly was performed using the Shovill pipeline, version 1.1.0 (https://github.com/tseemann/shovill), and the St. Petersburg genome Assembler (SPAdes), version 3.15.5 (7). Genome annotation was performed using PGAP, version 6.7 (8). The assembly (Table 1) was checked for plasmids with PlasmidFinder, version 2.0.1 (9), but no plasmids were found.

**TABLE 1:** Genomic features and accession numbers of *Pseudovibrio* sp.^*a*^

Isolate name	Genome size (bp)	Average coverage	No. of CDS	No. of rRNAs	No. of tRNAs	G + C content (%)	No. of contigs	*N*_50_ (bp)	*L*_50_	Assembly accession no.	SRA accession no.	No. of reads	Average read length (bp)
SCP19^*b*^	5,167,238	31.4	4,671	8	70	52.5	33	892,587	2	JBDZYJ000000000	SRR28745652	12,626,488	151

^*a*^ CDS, coding DNA sequences; SCP, Singapore coral probiotic.

^*b*^ SCP, Singapore coral probiotic.

Identification of the isolate was performed on the Microbial Genomes Atlas (10) web server using the NCBI-Prok function, with the closest homology to *Pseudovibrio* sp. FO-BEG1 based on average amino acid identity. Phylogenetic relatedness between the isolate and all publicly available *Pseudovibrio* genomes was determined using both the Orthologous Average Nucleotide Identity Tool, version 0.93.1 (11) and the Genome-To-Genome Distance Calculator, version 3.0 (12), to calculate the average nucleotide identity (ANI) and DNA-DNA hybridization (DDH) scores, respectively. Results showed a >95% ANI score with *Pseudovibrio denitrificans* DSM 17465 but did not share ≥70% DDH (species threshold) (13) with any *Pseudovibrio* genomes.

*Pseudovibrio* sp. SCP19 (for Singapore coral probiotics) produced brown colonies, and its genome encoded genes for the core enzymes of TDA production (*tdaABCDEF*) (5). The genome also encoded a dimethylsulfoniopropionate (DMSP) lyase gene (*dddD*) (14), indicating a potential to cleave DMSP into the cloud nucleating agent dimethylsulfide (15) and 3-hydroxypropionate, which can be converted into acrylate (16), the anti-herbivory compound (17). Analysis of the genome using GhostKOALA, version 2.0 (18), revealed the complete pathway for denitrification, thereby having the potential to reduce nitrates and nitrites into dinitrogen (19). These characteristics support the hypothesis that *Pseudovibrio* sp. SCP19 is a strong probiotic candidate.

## Data Availability

The SRA data were deposited to the National Center for Biotechnology Information, and the complete genome sequences were deposited to GenBank under the accession numbers listed in Table 1.

## References

[1] Goldberg SR, Haltli BA, Correa H, Kerr RG. 2022. Pseudovibrio flavus sp. nov. isolated from the sea sponge Verongula gigantea. Int J Syst Evol Microbiol 72:005457. doi:10.1099/ijsem.0.005457 35917228

[2] Parte AC, Sardà Carbasse J, Meier-Kolthoff JP, Reimer LC, Göker M. 2020. List of prokaryotic names with standing in nomenclature (LPSN) moves to the DSMZ. Int J Syst Evol Microbiol 70:5607–5612. doi:10.1099/ijsem.0.004332 32701423 PMC7723251

[3] Raina JB, Tapiolas D, Motti CA, Foret S, Seemann T, Tebben J, Willis BL, Bourne DG. 2016. Isolation of an antimicrobial compound produced by bacteria associated with reef-building corals. PeerJ 4:e2275. doi:10.7717/peerj.2275 27602265 PMC4994080

[4] Bondarev V, Richter M, Romano S, Piel J, Schwedt A, Schulz-Vogt HN. 2013. The genus Pseudovibrio contains metabolically versatile bacteria adapted for symbiosis. Environ Microbiol 15:2095–2113. doi:10.1111/1462-2920.12123 23601235 PMC3806328

[5] Henriksen NNSE, Lindqvist LL, Wibowo M, Sonnenschein EC, Bentzon-Tilia M, Gram L. 2022. Role is in the eye of the beholder-the multiple functions of the antibacterial compound tropodithietic acid produced by marine Rhodobacteraceae. FEMS Microbiol Rev 46:fuac007. doi:10.1093/femsre/fuac007 35099011 PMC9075582

[6] Bolger AM, Lohse M, Usadel B. 2014. Trimmomatic: a flexible trimmer for Illumina sequence data. Bioinformatics 30:2114–2120. doi:10.1093/bioinformatics/btu170 24695404 PMC4103590

[7] Bankevich A, Nurk S, Antipov D, Gurevich AA, Dvorkin M, Kulikov AS, Lesin VM, Nikolenko SI, Pham S, Prjibelski AD, Pyshkin AV, Sirotkin AV, Vyahhi N, Tesler G, Alekseyev MA, Pevzner PA. 2012. SPAdes: a new genome assembly algorithm and its applications to single-cell sequencing. J Comput Biol 19:455–477. doi:10.1089/cmb.2012.0021 22506599 PMC3342519

[8] Tatusova T, DiCuccio M, Badretdin A, Chetvernin V, Nawrocki EP, Zaslavsky L, Lomsadze A, Pruitt KD, Borodovsky M, Ostell J. 2016. NCBI prokaryotic genome annotation pipeline. Nucleic Acids Res 44:6614–6624. doi:10.1093/nar/gkw569 27342282 PMC5001611

[9] Carattoli A, Zankari E, García-Fernández A, Voldby Larsen M, Lund O, Villa L, Møller Aarestrup F, Hasman H. 2014. In silico detection and typing of plasmids using PlasmidFinder and plasmid multilocus sequence typing. Antimicrob Agents Chemother 58:3895–3903. doi:10.1128/AAC.02412-14 24777092 PMC4068535

[10] Rodriguez-R LM, Gunturu S, Harvey WT, Rosselló-Mora R, Tiedje JM, Cole JR, Konstantinidis KT. 2018. The microbial genomes atlas (MiGA) webserver: taxonomic and gene diversity analysis of Archaea and bacteria at the whole genome level. Nucleic Acids Res 46:W282–W288. doi:10.1093/nar/gky467 29905870 PMC6031002

[11] Lee I, Ouk Kim Y, Park SC, Chun J. 2016. OrthoANI: an improved algorithm and software for calculating average nucleotide identity. Int J Syst Evol Microbiol 66:1100–1103. doi:10.1099/ijsem.0.000760 26585518

[12] Meier-Kolthoff JP, Carbasse JS, Peinado-Olarte RL, Göker M. 2022. TYGS and LPSN: a database tandem for fast and reliable genome-based classification and nomenclature of prokaryotes. Nucleic Acids Res 50:D801–D807. doi:10.1093/nar/gkab902 34634793 PMC8728197

[13] Goris J, Konstantinidis KT, Klappenbach JA, Coenye T, Vandamme P, Tiedje JM. 2007. DNA-DNA hybridization values and their relationship to whole-genome sequence similarities. Int J Syst Evol Microbiol 57:81–91. doi:10.1099/ijs.0.64483-0 17220447

[14] Todd JD, Rogers R, Li YG, Wexler M, Bond PL, Sun L, Curson ARJ, Malin G, Steinke M, Johnston AWB. 2007. Structural and regulatory genes required to make the gas dimethyl sulfide in bacteria. Science 315:666–669. doi:10.1126/science.1135370 17272727

[15] Charlson RJ, Lovelock JE, Andreae MO, Warren SG. 1987. Oceanic phytoplankton, atmospheric sulphur, cloud albedo and climate. Nature 326:655–661. doi:10.1038/326655a0

[16] Todd JD, Curson ARJ, Nikolaidou-Katsaraidou N, Brearley CA, Watmough NJ, Chan Y, Page PCB, Sun L, Johnston AWB. 2010. Molecular dissection of bacterial acrylate catabolism--unexpected links with dimethylsulfoniopropionate catabolism and dimethyl sulfide production. Environ Microbiol 12:327–343. doi:10.1111/j.1462-2920.2009.02071.x 19807777

[17] Wolfe GV, Steinke M, Kirst GO. 1997. Grazing-activated chemical defence in a unicellular marine alga. Nature 387:894–897. doi:10.1038/43168

[18] Kanehisa M, Sato Y, Morishima K. 2016. BlastKOALA and GhostKOALA: KEGG tools for functional characterization of genome and metagenome sequences. J Mol Biol 428:726–731. doi:10.1016/j.jmb.2015.11.006 26585406

[19] Rädecker N, Pogoreutz C, Voolstra CR, Wiedenmann J, Wild C. 2015. Nitrogen cycling in corals: the key to understanding holobiont functioning? Trends Microbiol 23:490–497. doi:10.1016/j.tim.2015.03.008 25868684

